# Comparative Genomic Analysis of *Mycobacterium tuberculosis* Drug Resistant Strains from Russia

**DOI:** 10.1371/journal.pone.0056577

**Published:** 2013-02-20

**Authors:** Elena N. Ilina, Egor A. Shitikov, Larisa N. Ikryannikova, Dmitry G. Alekseev, Dmitri E. Kamashev, Maja V. Malakhova, Tatjana V. Parfenova, Maxim V. Afanas’ev, Dmitry S. Ischenko, Nikolai A. Bazaleev, Tatjana G. Smirnova, Elena E. Larionova, Larisa N. Chernousova, Alexey V. Beletsky, Andrei V. Mardanov, Nikolai V. Ravin, Konstantin G. Skryabin, Vadim M. Govorun

**Affiliations:** 1 Research Institute of Physical-Chemical Medicine, Moscow, Russian Federation; 2 Central TB Research Institute of RAMS, Moscow, Russian Federation; 3 Centre Bioengineering RAS, Moscow, Russian Federation; 4 Moscow Institute of Physics and Technology, Dolgoprudny, Russian Federation; Institut de Génétique et Microbiologie, France

## Abstract

Tuberculosis caused by multidrug-resistant (MDR) and extensively drug-resistant (XDR) *Mycobacterium tuberculosis* (MTB) strains is a growing problem in many countries. The availability of the complete nucleotide sequences of several MTB genomes allows to use the comparative genomics as a tool to study the relationships of strains and differences in their evolutionary history including acquisition of drug-resistance. In our work, we sequenced three genomes of Russian MTB strains of different phenotypes – drug susceptible, MDR and XDR. Of them, MDR and XDR strains were collected in Tomsk (Siberia, Russia) during the local TB outbreak in 1998–1999 and belonged to rare KQ and KY families in accordance with IS6110 typing, which are considered endemic for Russia. Based on phylogenetic analysis, our isolates belonged to different genetic families, Beijing, Ural and LAM, which made the direct comparison of their genomes impossible. For this reason we performed their comparison in the broader context of all *M. tuberculosis* genomes available in GenBank. The list of unique individual non-synonymous SNPs for each sequenced isolate was formed by comparison with all SNPs detected within the same phylogenetic group. For further functional analysis, all proteins with unique SNPs were ascribed to 20 different functional classes based on Clusters of Orthologous Groups (COG). We have confirmed drug resistant status of our isolates that harbored almost all known drug-resistance associated mutations. Unique SNPs of an XDR isolate CTRI-4^XDR^, belonging to a Beijing family were compared in more detail with SNPs of additional 14 Russian XDR strains of the same family. Only type specific mutations in genes of repair, replication and recombination system (COG category L) were found common within this group. Probably the other unique SNPs discovered in CTRI-4^XDR^ may have an important role in adaptation of this microorganism to its surrounding and in escape from antituberculosis drugs treatment.

## Introduction

*Mycobacterium tuberculosis* (MTB) is one of the most harmful worldwide human pathogens responsible for nearly 2 million deaths and 8–10 million new cases per annum [1]. In Russia, the epidemiological situation with tuberculosis (TB) is very alarming. In accordance with official statistics in 2009, the TB incidence rate was found to be 82.3 per 100000 population, and mortality rate was 2.8 per 100000 [2]. Almost 16% of newly diagnosed TB cases belonged to multidrug-resistant (MDR) TB, i.e. resistant at least to rifampicin and isoniazid (and 42.4% MDR among previously treated TB cases) [1]. The most complicated challenge is that the recently described extensively drug-resistant (XDR) TB was also reported in Russia [3]–[5].

Sequencing of the whole genomic DNA of MTB strain H37Rv in 1998 [6] provided a breakthrough in tuberculosis research, opening the way to understanding the biology, metabolism and evolution of this pathogen. Now, the development of new generation sequencing technologies reduces the cost and time required for genome sequencing, leading to the increasing availability of the whole genome sequences of microbial pathogens [7]. In recent years an increasing number of works brings us closer to the explanation of XDR TB formation. In contrast to MDR TB, molecular basis of which is well described [8], [9], the origin of XDR phenotype is unclear. There were various attempts devoted to discovery and detailed analysis of canonical and novel molecular mechanisms responsible for drug resistance [8], [10]–[15], to identification of essential genes for MTB growth and survival [16], to clarification of morphological and physiological differences between drug susceptible, MDR and XDR *M. tuberculosis* strains [17], [18]. A comprehensive analysis of the whole genome sequences (WGS) of a number of MTB isolates seems to be very useful for such investigations.

The first sequenced XDR strains were obtained from the KwaZulu-Natal (KZN) region of South Africa, isolated from a most notable outbreak of XDR-TB in 2005 [19]–[21]. Of these, the KZN-605 genome sequence is complete and annotated (according to the information from www.broadinstitute.org), while KZN-R506 strain has been sequenced up to 99% completion allowing an accurate detection of features (first at all, polymorphisms) relevant to the drug resistance [20], [22]. To date, a huge WGS data on MTB has been uploaded into the NCBI database, including the genomic information of XDR MTB strains isolated in Samara region, Russia [23].

In this study, we performed a genome sequencing of two drug-resistant clinical MTB isolates, which were isolated during a large tuberculosis outbreak in 1998–1999 in Tomsk (Siberia, Russian Federation), a region where Institutions of a Russian penitentiary system are located. In accordance with retrospective studies it was the first place in Russia where the XDR TB cases were described [3], [24]. Both selected strains showed the resistant phenotypes (CTRI-3^MDR^ and CTRI-4^XDR^, respectively). Basing on IS6110 RFLP DNA fingerprinting, they belonged to the KQ and KY families of MTB, respectively (according to the database of the Public Health Research Instutute, NJ, USA). These families are also known as Ural and Beijing respectively, and appear to be endemic to Russia [3], [25], [26].

Since no drug susceptible isolates of the above outbreak in Tomsk were available the drug susceptible isolate *M. tuberculosis* CTRI-2^SENS^ from Central region of the European Russia was included as an outgroup. This strain belonged to the AI (or LAM) family, which is also prevalent in Russia [27].

We hope that WGS of these *M. tuberculosis* strains could be one more step in filling the gap in the knowledge on a history and evolution of this pathogen, and finally will assist in understanding of the origin and possible ways of development of drug resistant MTB.

## Methods

### Strains, DNA Preparation and Drug Susceptibility Testing

Basic characteristics of the MTB isolates selected for current genome re-sequencing projects are presented in Table 1. The isolates under investigation were re-cultured from the laboratory bank in Dubos Broth supplemented with 5% BSA. Genomic DNA of MTB was extracted by the “Proba-NK” kit (“DNA-technology” Ltd. TY 9398-01646482062-2008, Russia) in accordance with the manufacturer’s instruction. The susceptibility testing of isolates was done using a BACTEC™ MGIT™ 960 Culture system (Becton Dickinson, USA) by standard protocol. All standard TB genotyping methods, including IS6110 RFLP, spoligotyping, and 24 loci MIRU-VNTR, were performed as previously described [28]–[30].

**Table 1:** Description of *M. tuberculosis* clinical isolates involved in this study.

Strain	type	Drug resistance^1^	IS6110 RFLP^2^	Spoligotypeclade^3^ (SIT)	24-VNTR^4^	SCG	Location
CTRI-2^SENS^	susceptible	RIF^S^, INH^S^, EMB^S^, STR^S^,PZA^S^, ETH^S^, AMI^S^, CAP^S^,OFL^S^	AI 114	LAM 9 (42)	124225153225323124123362	5	Vladimir(Central region)
CTRI-3^MDR^	MDR	RIF^R^, INH^R^, EMB^R^, STR^R^,PZA^R^, AMI^R^, CAP^R^;ETH^S^,OFL^S^	KQ	Haarlem4/Ural-1^5^ (262)	227125113224353244423463	3a	Tomsk (Siberia)
CTRI-4^XDR^	XDR	RIF^R^, INH^R^, EMB^R^, STR^R^,PZA^R^, ETH^R^, AMI^R^, CAP^R^,OFL^R^	KY	Beijing-like (269)	223325173423254644423373	2	Tomsk (Siberia)

1 RIF - rifampicin, INH - isoniazid, EMB - ethambutol, STR - streptomycin, PZA - pyrazinamide, ETH - ethionamide, AMI- amikacin, CAPR - capreomycin, OFL - ofloxacin.

2 according PHRI TB Centre database.

3 SpolDB4 was used for identification of data [54].

4 24– VNTR: MIRU 02, MIRU 04, MIRU 10, MIRU 16, MIRU 20, MIRU 23, MIRU 24, MIRU 26, MIRU 27, MIRU 31, MIRU 39, MIRU 40, VNTR 42, VNTR 43, VNTR 1955, QUB-11b, ETRA, VNTR 46, VNTR 47, VNTR 48, VNTR 49, VNTR 3690, QUB-26, VNTR 53 [29].

5 Ural-1 subfamily in accordance with Mokrousov [38].

### Genome Sequencing and Assembly

Genomes were sequenced on a Roche Genome Sequencer GS FLX using a standard protocol for a shotgun genome library. Assembly of raw sequencing reads with an average length of 215 bases was performed by the GS de novo assembly software version 1.1.03.24 (Roche 454 Life Science). In particular, the order of contigs was predicted by comparison with full-length genomes: H37Rv, H37Ra, F11, CDC1551. Mauve 2.3.1 was used for visualization of the data [31].

To determine the full-length genomic sequence of CTRI-2^SENS^, the regions between contigs corresponding to repetitive regions or regions with no coverage were additionally sequenced on ABI Prism® 3730 Genetic Analyzer («Applied Biosystems», USA; «Hitachi», Japan).

### Sequence Analysis and Annotation

In order to facilitate a comparison to previous large-scale studies and to facilitate further study of the SNPs (single nucleotide polymorphisms) determined in this investigation, we computed SNPs in a number of closely related mycobacterial genomes relative to H37Rv (AL123456.2). It was done using MUMmer 3.20 with its nucmer and show-snps functions [32] for *M. tuberculosis* strains CDC1551 (AE000516.2), F11 (CP000717.1), KZN-4207 (CP001662), C (AAKR00000000), HN878(ADNF00000000.1), T85 (ABOW00000000), 210 (ADAB00000000), CCDC5079 (CP001641), KZN-1435 (CP001658.1), KZN-V2475 (ACVT00000000), R1207 (ADNH00000000.1),W-148 (ACSX00000000.1), X122 (ADNG00000000.1), KZN-R506 (ACVU00000000), KZN 605 (ABGN00000000), Haarlem (AASN00000000), CCDC5180 (CP001642), 02_1987 (ABLM00000000), 94_M4241A (ABLL00000000), EAS054 (ABOV00000000), T46 (ACHO00000000) (Table 2).

**Table 2:** Description of *M. tuberculosis* strains considered under current investigation.

No	*M. tuberculosis* genome(GenBank Accession Number)	Type^1^	Spoligotypeclade^2^	Location	Sequenced by
1	H37Rv (AL123456.2)	DS	H37Rv		Welcome Trust Sanger Institute, 1998, complete
2	CDC1551 (AE000516.2)	DS	X3	USA	TIGR, 2002, complete
3	F11 (CP000717.1)	DS	LAM3	Western Cape of South Africa	Broad Institute, 2007, complete
4	3KZN-4207 (CP001662)	DS	LAM4	KwaZulu-Natal, South Africa	Texas A&M University, in progress
5	CTRI-2^SENS^ (CP002992)	DS	LAM9	Vladimir, Russia (Central region)	this study, 2010, complete
6	C (AAKR00000000)	DS	NA	NA	Broad Institute, in progress
7	HN878 (ADNF00000000.1)	DS	Beijing (modern)	USA	Texas A&M University, in progress
8	T85 (ABOW00000000)	DS	Beijing (modern)	Isolated in San Franciscoin 1998 from a patient bornin China	Broad Institute, in progress
9	210 (ADAB00000000)	DS	Beijing (modern)	Los Angeles,USA	TIGR, in progress
10	CCDC5079 (CP001641)	DS	Beijing (modern)	Fujian Province, China	Beijing Genomics Institute, complete
11	CTRI-3^MDR^(SRA051492)	MDR	Haarlem4/Ural-1^4^	Tomsk, Russia (Siberia)	this study, in progress
12	KZN-1435 (CP001658.1)	MDR	LAM4	KwaZulu-Natal, South Africa	Broad Institute, 2009, complete
13	KZN-V2475 (ACVT00000000)	MDR	LAM4	KwaZulu-Natal, South Africa	Texas A&M University, in progress
14	R1207 (ADNH00000000.1)	MDR	Beijing (ancient)	Western Cape of South Africa	Texas A&M University, in progress
15	W-148 (ACSX00000000.1)	MDR	Beijing (modern)	Russia	Broad Institute, in progress GenBank:
16	X122 (ADNG00000000.1)	pre-XDRstrain^5^	Beijing (modern)	Western Cape of South Africa	Texas A&M University, in progress
17	CTRI-4^XDR^(AIIE01000000)	XDR	Beijing (ancient)	Tomsk, Russia (Siberia)	this study, in progress
18	3KZN-R506 (ACVU00000000)	XDR	LAM4	KwaZulu-Natal, South Africa	Texas A&M University, in progress
19	KZN 605 (ABGN00000000)	XDR	LAM4	KwaZulu-Natal, South Africa	Broad Institute, 2010, complete
20	Haarlem (AASN00000000)	DR	H	The Netherlands	Broad Institute, complete
21	CCDC5180 (CP001642)	DR	Beijing (modern)	Fujian Province, China	Beijing Genomics Institute, complete
22	02_1987 (ABLM00000000)	NA	Beijing (ancient)	Isolated in San Franciscoin 2002 from a patient bornin South Korea	Broad Institute, in progress
23	94_M4241A (ABLL00000000)	NA	Beijing (ancient)	Isolated in San Franciscoin 1994 from a patient bornin China	Broad Institute, in progress
24	EAS054 (ABOV00000000)	NA	EAI	Isolated in San Franciscoin 1993 from a patient bornin India	Broad Institute, in progress
25	T46 (ACHO00000000)	NA	EAI	Isolated in San Franciscoin 1996 from a patient born inThe Philippines	Broad Institute, in progress

1 NA not available, DS – drug susceptible, DR – drug resistant, MDR - multidrug resistant, XDR - extensively drug-resistant.

2 SpolDB4 was used for identification of data [54].

3 Full-length genome sequence in which regions with zero coverage filled by nucleotides from the reference genome F11 are also available [20].

4 Ural-1 subfamily in accordance with Mokrousov [38].

5 It is resistant to isoniazid, rifampicin, and ofloxacin, and it is susceptible to ethionamid, amikacin, and ethambutol.

To search for genomic rearrangements in strain CTRI-2^SENS^ relative to H37Rv, MUMmer 3.20 with its run-mummer3 algorithm was used for full alignment of sequences.

Annotation of coding sequences for isolate CTRI-2^SENS^ was completed by comparison to RefSeq annotation of *M. tuberculosis* strain H37Rv. Insertion sequences that were not present in the genome of H37Rv were annotated by comparison with other mycobacteria.

### Multilocus Sequence/SNPs Analysis for Phylogeny

25 partially- or whole-genome sequenced MTB strains, which included both three strains studied and 22 genomes uploaded into the NCBI database (Table 2) were scanned for on the nucleotide polymorphisms in different loci of genome.

At first, all SNPs (refer to H37Rv) were extracted from genomes and used for the phylogenetic tree building. Next, we have analyzed 42 core genes on the nucleotide diversity index. Of 42, eight genes were taken away as non-variable, six were excluded because of poor quality of sequences, and in two cases the dN/dS ratio exceeded 1.0. Twenty six loci were used for the analysis of phylogenetic relationships. Furthermore, a set of DNA repair, replication and recombination (3R) genes was delineated in accordance with Dos Vultos et al. [33] and utilized for the designation of phylogenetic relationships.

BioEdit v.7.0.9.0, and MEGA v.4.0 were used for examination of sequences and phylogenetic evolutionary analysis. DNAsp v.5.10 was used for the estimation of d_N_/d_S_ ratios and the nucleotide diversity of genes.

## Results

### Strains

Clinical isolates of MTB obtained from patients with newly diagnosed pulmonary tuberculosis have been collected in Central Tuberculosis Research Institute (Moscow, Russia) in 1999. All isolates were tested for the susceptibility to first-line antituberculosis drugs and typed for IS6110 RFLP, and then stored in the laboratory bank.

In 2007, these isolates were subcultured and re-tested on the susceptibility to the first-line and second-line drugs including fluoroquinolone, prior to genome sequencing. Isolate CTRI-2^SENS^ was confirmed as drug-susceptible, while two isolates, CTRI-3^MDR^ and CTRI-4^XDR^, were characterized as MDR and XDR, respectively. Later, the IS6110 RFLP, spoligotyping, and VNTR analysis were additionally performed (Table 1).

### Genome Sequencing Data

Genomes of the three isolates were sequenced to 98% completion using 454 Whole Genome Shotgun methodology with greater than 10-fold of coverage. The GS De Novo Assembler software was used to assemble the reads, obtaining at a first step 517, 573 and 739 contigs with an N50 length of 18425, 13668 and 11896 bp for CTRI-2^SENS^, CTRI-3^MDR^ and CTRI-4^XDR^, respectively. However, mycobacterial genomes contain a lot of repetitive elements, as well as poorly readable regions of high G+C content, so in some cases we additionally used a Sanger sequencing instrumentation to get poor covered fragments.

The resulting (circular) genome of CTRI-2^SENS^ was obtained (GenBank accession number is CP002992). It has been composed by 4398525 nucleotides and characterized with a G+C content of 65.6%. At least, 955 isolated SNPs referring to the H37Rv genome were revealed: 862 of 955 SNPs occurred in coding regions; 324 and 538 of them were identified as synonymous (sSNPs) and non-synonymous (nsSNPs) SNPs, respectively. Seven of these nsSNPs were nonsense mutations. 247 of 862 SNPs were common for all genomes included in our research, so we may postulate these polymorphisms as specific for H37Rv.

One set of rRNAs and 45 tRNA genes were identified in the CTRI-2^SENS^ genome, as well as 3946 protein-coding genes with the average gene length of 1004 bp. In total, protein-coding genes represent 90.3% of whole genome. More than 97% of coding sequences mapped to H37Rv genes.

Also, 204 indels were found in CTRI-2^SENS^ with respect to H37Rv genome. More details are available in Supplement file (Text S1).

**Figure 1:**
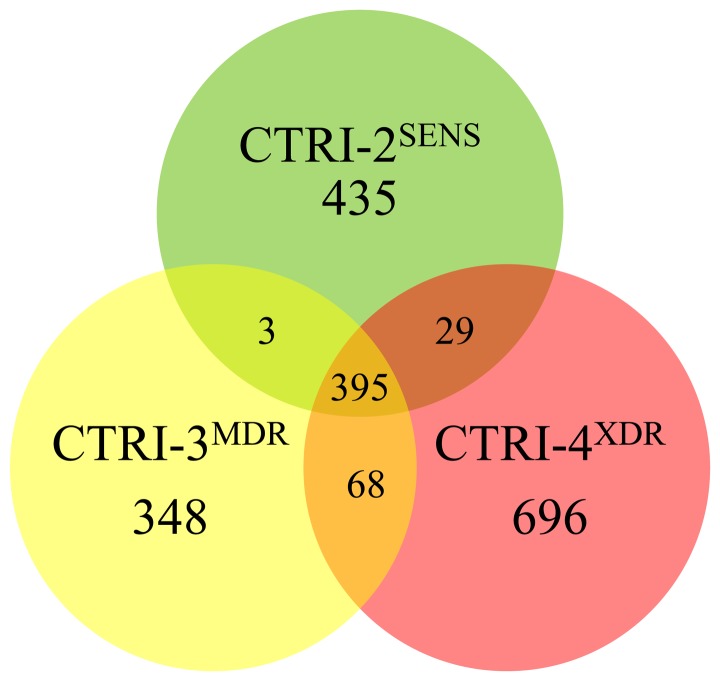
Venn diagram of single nucleotide polymorphisms in three studied isolates.

The sequences of CTRI-3^MDR^ and CTRI-4^XDR^ isolates were obtained in a similar way. 814 SNPs in CTRI-3^MDR^ and 1188 SNPs in CTRI-4^XDR^ belonged to coding regions. Venn diagram showing the SNPs distribution in three genomes under study compared with the H37Rv demonstrates that all our strains appeared to be quite different (Figure 1): only 395 polymorphisms are common, while each isolate possessed a considerable number of individual SNPs.

### Genetic Markers of Drug Resistance

For studied MDR and XDR MTB isolates the pattern of mutations conferring the drug resistance was determined. According to our analysis, we found almost all genetic markers reflecting the certain susceptibility phenotype (Table 3). We also looked for compensatory mutations in *rpoA* and *rpoC* genes described recently [34], and did not find any of them in studied strains.

**Table 3:** Polymorphisms in CTRI-3^MDR^ and CTRI-4^XDR^ strains associated with drug resistance.

Drug resistance effect	Gene	CTRI-3^MDR^	CTRI-4^XDR^	Reference
Rifampicin (RIF)	*rpoB* 1	Asp435Val (Asp516Valin *E. coli* numbering)	Asp435Val (Asp516Valin *E. coli* numbering)	[66]–[69]
Isoniazid (INH)	*katG* 1	Ser315Thr	Ser315Thr	[70]–[72]
Ethambutol (EMB)	*embB* 1	Met306Ile	Met306Val	[73], [74]
	*embB* 2	Pro832Ser		[73], [74]
Pyrazinamide (PZA)	*pncA* 1		His51Pro	[75]
	*rpsA* 2		Asp123Ala	[76]
Kanamycin(KAN)/Amikacine(AMI)/Streptomycin (STR)	*rrs* 1	A1401G		[77]–[79]
	*rpsL* 1		Lys88Arg	[80], [81]
Ethionamide (ETH)	*ethA* 2		ΔA1345	[82]
	*ndhA* 2		His164Gln	[83]
Fluoroquinolone (FQ)	*Rv0194* 2		Pro1098Leu	[35]
	*Rv1634* 2		Gly198Arg	[37]
	*Rv2688c* 2		Cys213Ard	[36]

1 canonical mutations associated with drug-resistance.

2 mutations probably associated with drug-resistance.

Unfortunately, mechanism of FQ resistance in CTRI-4^XDR^ isolate remains unclear but probably efflux pumps might be involved. We have checked out mutations in known genes coding the probable membrane transporters and efflux pumps [35] and found that CTRI-4^XDR^ (in contrast to CTRI-3^MDR^) carried some dissimilar amino acid substitutions in drugs-transport transmembrane ATP-binding protein ABC transporter Rv0194 (Pro1098Leu), drug efflux membrane protein Rv1634 (Gly198Arg), antibiotic ABC transporter ATP-binding protein Rv2688c (Cys213Arg).

In former studies, the Rv2686c-Rv2687c-Rv2688c proteins were found to be responsible for active efflux of FQs outside the bacterial cell [36], and the expression of Rv1634 was found to confer a low level of FQs resistance [37]. Although there is no data how mutations in these particular proteins can effect on drug resistance, we suspect that they could have a cumulative effect on formation of FQ resistance in case of CTRI-4^XDR^.

### Phylogenomic Analysis

In accordance with IS6110 RFLP typing, CTRI-2^SENS^ *M. tuberculosis* isolate contained 13 copies of IS6110 and was referred to AI114 family. The drug-resistant isolates, CTRI-3^MDR^ and CTRI-4^XDR^, contained 12 and 10 copies of IS6110, respectively, and were attributed to the KQ and KY families of MTB, which were found in the last years of XX century and are considered to be endemic for Russia [3], [25], [26].

Analysis of the direct-repeat (DR) region indicated that CTRI-2^SENS^, CTRI-3^MDR^, and CTRI-4^XDR^ belonged to LAM9, Haarlem4, and Beijing families according to SpolDB4, respectively. In accordance with Mokrousov work CTRI-3^MDR^ should be referred to Ural-1 family [38]. It should be noted that CTRI-4^XDR^ had abridged Beijing-like profiles with 35 and 36 signals absent and belongs to the ancient/ancestral sublineage [39], [40].

The obtained WGS were used to determine the SNP Cluster Groups (SCGs) [41] for analyzed isolates, which were found SCG5, SCG3a and SCG2 for CTRI-2^SENS^, CTRI-3^MDR^ and CTRI-4^XDR^, respectively. Also, we defined the principal genetic groups (PGG) based on the determination of nucleotide polymorphisms in two codons of *katG* (katG463) and *gyrA* (gyrA95) genes [42], which were found PGG2 for both CTRI-2^SENS^ and CTRI-3^MDR^, in contrast to PGG1 for CTRI-4^XDR^.

The 25 of MTB whole- or partially-genome sequences were drawn for the comparative phylogenetic analysis. In addition to three isolates under study (CTRI-2^SENS^, CTRI-3^MDR^ and CTRI-4^XDR^), this group included twenty two genomes presented in the NCBI database (Table 2).

**Figure 2:**
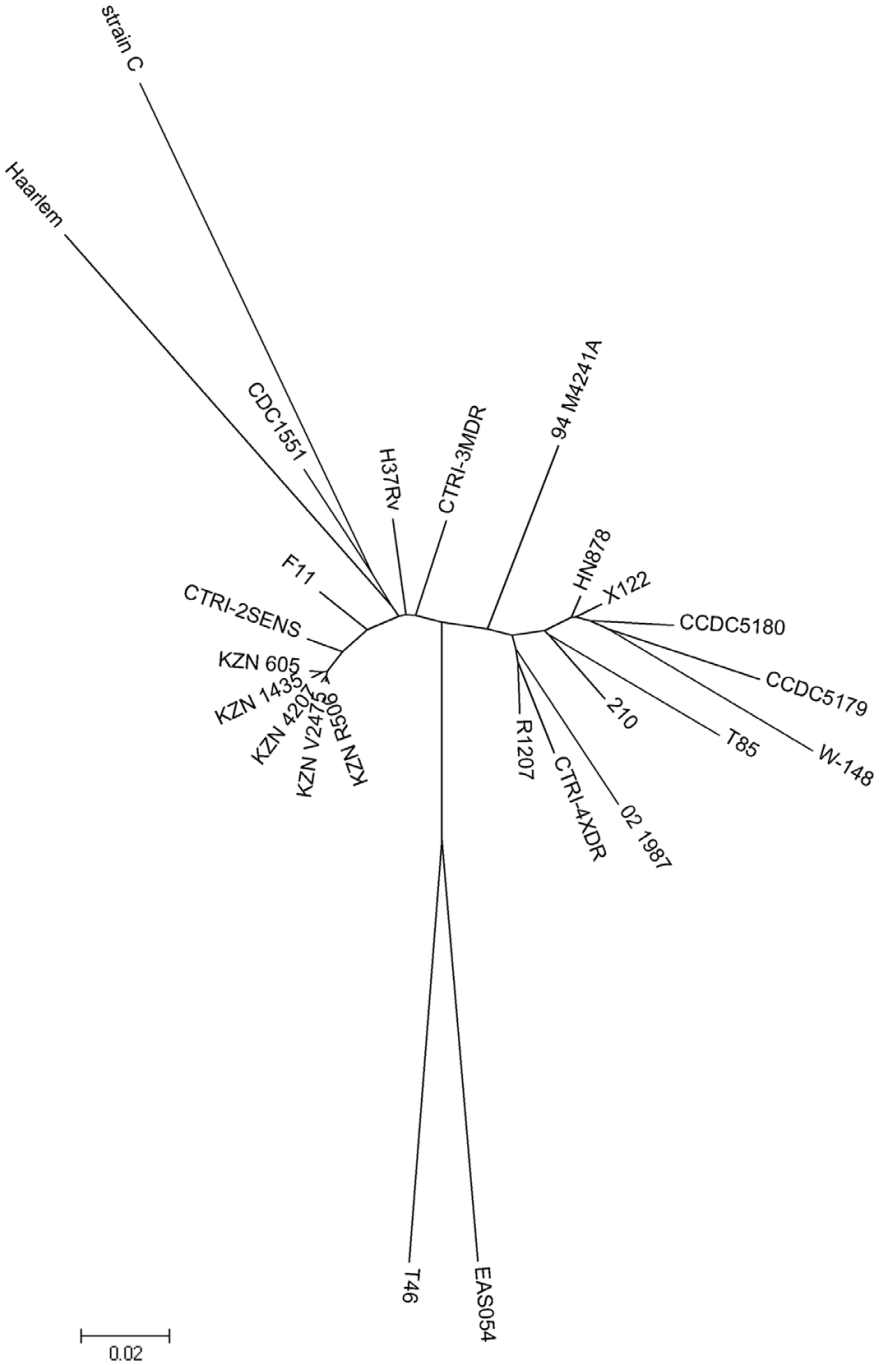
Comparative phylogenetic analysis of strains under study and 22 whole genomes from the NCBI database. Tree based on all SNPs of genomes. Phylogenetic tree was constructed using the Neighbor-Joining algorithm. Evolutionary distances were calculated using p-distance method. The bootstrap test was for 1000 replicates. Branches corresponding to partitions reproduced in less than 50% bootstrap replicates are collapsed.

Phylogenetic tree was built based on overall SNPs extracted from genomic DNA sequences. Such way does not give the true phylogenetic relationships in a case of quickly evolving microorganisms disposed to the recombination; however, it can be very effective in respect of the genetically monomorphic bacteria like *M. tuberculosis* [43]. This approach divided all isolates into different groups, establishing the similarity of our drug susceptible strain, CTRI-2^SENS^, and F11, and, also, KZN-like strains; of CTRI-3^MDR^ and CDC1551, Haarlem and C; of CTRI-4^XDR^ and 02-1987, 94_M4241A, X122, W-148, R1207, HN878, T85, 210, CCDC5079 and CCDC5180 strains (“CTRI-4^XDR^ cluster”) (Figure 2). Additionally, phylogenetic tree was constructed using a set of SNPs in 26 housekeeping loci (*aroA, atpD, dnaK, ffh, ftsK, fusA1, gcvB, glnA1, groEL2, gyrA, gyrB, inhA, katG, leuS, lysA, polA, recA, rplA, rplD, rplE, rplM, rplN, rpsK, secA1, thyA and topA*), and 75 genes encoding 3R system components of MTB: 56 loci designated in the work of Dos Vultos et al. [33] and 19 additional loci – *dnaE, fpg, dnaG, dnaA, dnaB, xerC,* Rv3202c, Rv3263, Rv3828c, *helY*, *tatD, xseA, priA, rnhB, ercc3, deaD, dinG, helZ* and *rhlE*. In all cases, we have got very similar strains’ distribution as the trees based on overall SNPs (data not shown).

### COGs Analysis

To study new molecular mechanisms probably associated with virulence and drug resistance formation of TB the list of unique individual SNPs was formed for each sequenced isolate. Whereas the studied isolates – drug-susceptible, MDR and XDR, appeared to be rather genetically heterogenic, we have considered only SNPs present in certain isolate and absent in other genetically related MTB strains. Along this line only full sequenced and well characterized strains were considered (Table 2). Thus for CTRI-2^SENS^ such genetically related strains were assigned as F11, KZN 1435, and KZN 605. For CTRI-3^MDR^, this group consisted of CDC1551 and Haarlem, and during the analysis of SNPs present in CTRI-4^XDR^ the ones from CCDC5079 and CCDC5180 were excluded.

In total, there were 127, 367, and 246 individual SNPs identified for CTRI-2^SENS^, CTRI-3^MDR^, and CTRI-4^XDR^ isolates, respectively (see Table S1, S2, and S3). All proteins carried these unique SNPs were ascribed to 20 different functional classes based on Clusters of Orthologous Groups (COG) [44], [45] that is also presented in supplementary tables.

CTRI-2^SENS^ individual SNPs were perfectly unique ones, without intersections with CTRI-3^MDR^ and CTRI-4^XDR^. And there were only two SNPs shared by CTRI-3^MDR^ and CTRI-4^XDR^: in Ser315 codon of *katG* gene (Ser315Thr) and in Asp435 (Asp516 in *E. coli* numbering) of *rpoB* gene (Asp435Val) conferring resistance to INH and RIF, respectively. There were 67.5% (166/246) individual SNPs identified as non-synonymous substitutions (assigned as single amino acid polymorphisms, SAPs) in CTRI-4^XDR^ isolate, and slightly fewer ones in CTRI-2^SENS^ and in CTRI-3^MDR^: 59.8% (76/127) and 58.6% (215/367), respectively.

**Figure 3:**
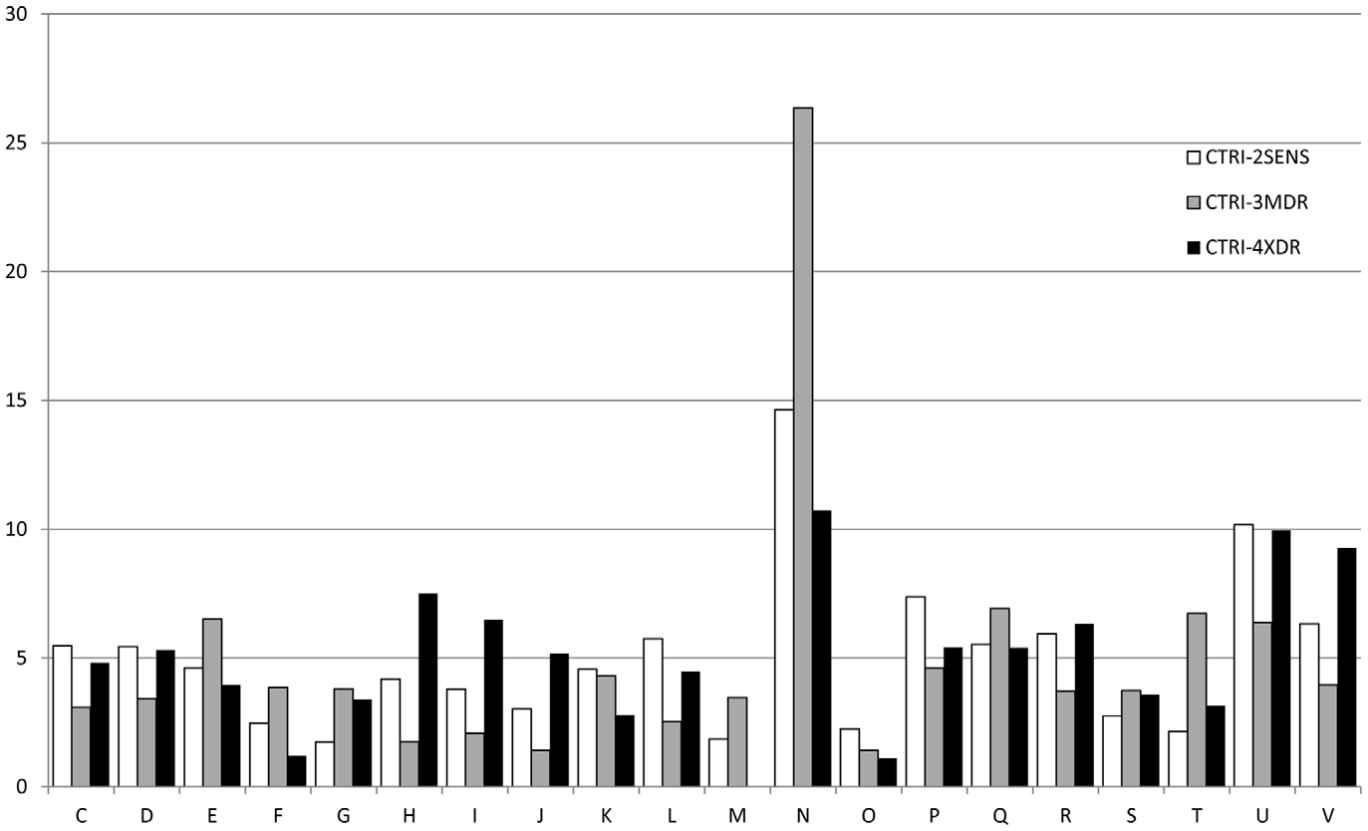
The relative distribution of individual SAPs found in proteins belonged to certain COGs. C - energy production and conversion; D - cell cycle control, cell division, chromosome partitioning; E - amino acid transport and metabolism; F - nucleotide transport and metabolism; G - carbohydrate transport and metabolism; H - coenzyme transport and metabolism, I - lipid transport and metabolism, J - translation, ribosomal structure and biogenesis; K – transcription; L - replication, recombination and repair; M - cell wall/membrane/envelope biogenesis, N - cell motility, O - posttranslational modification, protein turnover, chaperones; P - inorganic ion transport and metabolism; Q - secondary metabolites biosynthesis, transport, and catabolism; R - general function prediction only; S - function unknown; T - signal transduction mechanisms; U - intracellular trafficking, secretion, and vesicular transport; V - defense mechanisms.

The distribution of individual SAPs in certain COGs was analyzed comparatively for three isolates. The number of SAPs was normalized with respect to the number of genes of a certain class present in MTB and then to the total number of individual SAPs identified in certain isolates (Figure 3). The calculated average number of SAPs was found 5.00, and a standard deviation was 3.81.

All isolates possessed very few SAPs mapped to O (posttranslational modification, protein turnover, chaperones) COG, suggesting that the genes in this category may be under evolutionary pressure. Additionally, the CTRI-4^XDR^ isolate had significantly fewer SAPs mapped to F (nucleotide transport and metabolism) COG than the other ones, and revealed no SAPs in proteins belonging to M (Cell wall/membrane/envelope biogenesis) group.

In all cases, the significant numbers of SAPs were found in proteins from N (cell motility) COG. It is not surprising because they belong to the PE/PPE protein family which reflects the natural variability of surface structures. One also can see the expose of the relative density of mutations in proteins of U (Intracellular trafficking, secretion, and vesicular transport) COG for CTRI-2^SENS^ and CTRI-4^XDR^, and of V (defense mechanisms) COG for CTRI-4^XDR^ only. The last observation may be directly related to the formation of the XDR phenotype.

In case of XDR tuberculosis, it was rather interesting to analyze other mutations, different from related to drug resistance ones, because they can play an important role in survival, adaptation and spreading of such strains in microbial population. Thus, the unique SAPs in CTRI-4^XDR^ were analyzed more precisely. Along this line, we reduced the full list of mutations by excluding PE/PPE protein family, hypothetical proteins, and also proteins with homogenous amino acid substitutions (Table S4). All SNPs presented in the table S4 were verified by Sanger sequencing.

As it might have been expected the known mutations in *rpoB*, *katG*, *pnzA*, *embB* and *rpsL* genes, conferring resistance to RIF, INH, PZA, EMB, and STR respectively, fell into this list. We also found a mutation in *thyA* gene, which leads to Asp117Gly amino acid substitution. Recently, the mutations in thymidylate synthase A, encoded by the *thyA* gene and required for a thymine biosynthesis in the folate pathway, were shown to be associated with para-aminosalicylic acid (PAS) resistance in MTB [46]. However, this particular Asp117Gly alteration was not described yet neither in clinical *M. tuberculosis* strains resistant to PAS nor in PAS-resistant spontaneous mutants [47]. It should be mentioned that susceptibility testing of the CTRI-4^XDR^ strain to this drug was not done either.

In contrast to CTRI-2^SENS^ and CTRI-3^MDR^, it is noteworthy that CTRI-4^XDR^ isolate exposed much more mutations in proteins belonging to the categories H (coenzyme transport and metabolism), I (lipid transport and metabolism), J (translation, ribosomal structure and biogenesis), and V (defense mechanisms) (Figure 1).

It is rather difficult to interpret mutations in J COG, mostly happened in ribosomal proteins, but the observed aberrations in proteins belonged to I and V classes along with mutant enzymes from C (energy production and conversion) COG may play an important role in adaptation of the microorganism to its surrounding. This way, we can speculate that some of the listed mutations might be related to survival of mycobacteria under stress, including the drug treatment. So we observed a lot of mutations in proteins belonged to oxidation-reduction enzymes - NADH dehydrogenase, enoyl-CoA hydratase, acyl-CoA dehydrogenases, electron transfer protein FdxB. Earlier, the disorder in functioning of a redox system was shown for some human cell lines revealed the resistance to anticancer drugs [48]. Aware of the illegitimacy of the parallels between pro- and eukaryotes we can suggest that some features of the internal MTB metabolism promote a better adaptation of these cells and create more comfortable conditions for the formation of resistance to antituberculosis agents. Perhaps the further contribution to this process makes changes in DNA polymerase III, one of the SOS response enzymes.

As far as mutations in ribosomal proteins are concerned, we can suspect their involvement in translation fidelity. Besides of substitutions in *rpsL* and *rpsA* genes associated with drug resistance that has been already noted above, we indicated Lys69Arg replacement in 30S ribosomal protein S17 encoding by *rpsQ* gene. It’s one of the primary rRNA binding proteins; it binds specifically to the 5′-end of 16S ribosomal RNA and also plays a role in translation fidelity. The altered form of this protein may affect the accuracy of translation, and thereby leads to errors in the process of protein synthesis.

Other features of CTRI-4^XDR^ genomic DNA sequences were mutations in ParA and RodA proteins, controlling certain stages of cell division. Since the expression of *parA* gene is essential for the growth of mycobacteria [49], discovery of a mutant protein in CTRI-4^XDR^ isolate allows us to explain the slightly slower accumulation of cell biomass in the liquid medium culture observed earlier. Perhaps recorded mutations lead to uncompleted cell division, previously described as a feature of XDR TB strains [17].

When we used the same algorithm to identify the unique mutations characteristic for XDR TB from KwaZulu-Natal region of South Africa (see Table S5), it became obvious that they have a little in common with CTRI-4^XDR^ with the exception of mutations in *pks* genes cording the polyketide synthases (we did not consider mutations in *rpo* gene). Although the different genes were found to be altered, *pks1* and *pks7* for CTRI-4^XDR^ and *pks12* for KZNs, their products both belonged to similar multifunctional enzymes each of which contains a β-ketoacyl synthase, and an acyltransferase activities (www.kegg.org). Generally, they catalyze polymerization of simple fatty acids into branched-chain ones. In spite of the MTB genome has revealed a remarkable array of polyketide synthases, no polyketide product has been isolated thus far. Most of the polyketide synthases genes have been implicated in the biosynthesis of complex lipids, and also in a lipopolysaccharide biosynthesis. In light of this one can assume that the wrong activity of these enzymes leads to disorders in cell wall structure and in its permeability for anti-tuberculosis agents.

### Occurrence of Selected for CTRI-4^XDR^ Isolate Mutations among the other Beijing MTB Strains

To solve the question if there are other MTB strains carried any mutations from the list formed for CTRI-4^XDR^ (Table S4) we marked out a group of eight Beijing TB strains (Table 2) which draft genomic sequences were publicly available from NCBI web site. Additionally, we tried to find these SNPs in WGS data for 26 MDR and 13 XDR *M. tuberculosis* strains from the work of Casali N. *et al* [23].

Throughout analysis revealed that the mutations in Rv0068 and Rv1266c genes should be assigned as polymorphisms because of their presence in the most of strains.

As well, we identified a large group of mutations (24 from 76; 31.5%) shared by CTRI-4^XDR^, 02_1987, and R1207 TB strains. All belonged to the Beijing sublineage [39], [40]. Taking into account that we were failed to find any information on 02_1987 phenotype, presumably it is a drug susceptible strain, while the R1207 is known to be MDR, our attention was mostly caught by five mutations in Rv0667 (*rpoB* ), Rv1589 (*bioB*), Rv1908c (*katG*), Rv1934c (*fadE17*) and Rv3158 (*nuoN*) genes shared by CTRI-4^XDR^ and R1207 only. Among them, the canonical mutations in *rpoB* and katG genes conferring the RIF and INH resistance are not so interesting, while the other ones in *fadE17*, and *nuoN* genes can support the drug resistance formation via the changes in functioning of a redox system. Besides CTRI-4^XDR^ as well as R1207 carried a mutant variant of an enoyl-CoA hydratase (*echA10*), another enzyme belonged to a redox system. However in this case, the amino acid substitutions were different.

To support the idea that some of these thought-out mutations of CTRI-4^XDR^ actually involved in formation of XDR phenotype; we have sequenced the additional XDR *M. tuberculosis* isolates (n = 14) collected in the different regions of Russian Federation in the period 2006–2010 years. Thirteen of them belonged to the modern Beijing group in accordance with spoligotyping and *mutT2* gene analysis [50].

All strains were sequenced for mutations in the following genes of interest: *nuoN, icd2, dnaE, rodA, parA, enhA1, enhA10, bioB, fadB3, fadE17*, *fdxB, rpsL* and *gyrA* (Table 4). The latter two were included to check of the potential XDR genotype. Nearly all strains possessed any mutations in Ala90 or/and Asp94 of GyrA, and Lys43Arg substitution in ribosomal protein S12 encoding by *rpsL* gene, which is different from CTRI-4^XDR^ strain. The other mutations we were looking for were absent in all additional strains, suggesting that CTRI-4^XDR^ evolved by its own individual way.

**Table 4:** Mutations of interest characteristic for CTRI-4^XDR^ isolate.

Gene	COG Group	Gene name	Product	aa substitution	dissimilarityindex^1^	shared with
Rv3158	C	*nuoN*	NADH dehydrogenase subunit N	Ala362Ser	1	R1207
Rv0066c	C	*icd2*	isocitrate dehydrogenase	Asp374Ala	−2	unique
Rv0017c	D	*rodA*	cell division protein RodA	Phe28Val	−1	unique
Rv3918c	D	*parA*	chromosome partitioning protein ParA	Ser138Phe	−2	unique
Rv1589	H	*bioB*	biotin synthase	Arg66Gly	−2	R1207
Rv0222	I	*echA1*	enoyl-CoA hydratase	Ala248Ser	1	unique
Rv1934c	I	*fadE17*	acyl-CoA dehydrogenase FADE17	Glu394Lys	1	unique
Rv1934c	I	*fadE17*	acyl-CoA dehydrogenase FADE17	Leu34Arg;	−2;	R1207
Rv1142c	I	*echA10*	enoyl-CoA hydratase	Lys59Arg	2	unique
Rv1715	I	*fadB3*	3-hydroxybutyryl-CoA dehydrogenase FADB3	Trp188Arg	−3	unique
Rv3554	I	*fdxB*	electron transfer protein FdxB	Val358Leu	1	unique
Rv0682	J	*rpsL*	30S ribosomal protein S12	Lys88Arg	2	unique
Rv1547	L	*dnaE*	DNA polymerase III subunit alpha	Ser898Leu	−2	R1207, 02_1987

1 Indicated in accordance with BLOSUM62 substitution matrix [84], [85].

## Discussion

In spite of the hard efforts of many researchers in the world to gain insight into the origin and nature of *M. tuberculosis* drug-resistant strains, it still remains obscure. In early XXI century, an attention of clinicians and scientists has been re-focused from the multidrug-resistant tuberculosis to the extensively drug-resistant one. Although the definition of XDR-TB was agreed by the WHO in October 2006 [51], from 1993 through 2006 forty-nine cases of XDR TB have just been reported in the United States [52]. Such strains, which show the resistance to the most of routinely used anti-tuberculosis drugs, are seriously hindering a successful TB control. Understanding the molecular basis of XDR/MDR phenotypes could help in the developing of new approaches to the diagnosis, treatment and control of this disease.

Today, against the background of the implementation of numerous genome projects, particularly in the genomics of microorganisms, the main expectations in understanding of the molecular mechanisms of virulence and resistance of pathogens, as well as in discovery new targets for chemotherapy confer on the interpretation of the WGS data.

Under this study, we hoped to come closer to understanding of the XDR phenotype formation mechanisms by means of comparative analysis of WGS data obtained for two drug-resistant isolates CTRI-3^MDR^ and CTRI-4^XDR^, attributed to the unique KY and KQ families of MTB by IS6110 RFLP DNA fingerprinting, presumably endemic for Russia. Additionally drug-susceptible isolate CTRI-2^SENS^ was introduced as an outgroup. Both resistant strains were selected from the collection of *M. tuberculosis* isolates gathered in Tomsk, Siberia, Russia, during the well-known outbreak in 1998–2002 years [3].

Earlier, the study of similar design was done for MTB strains isolated in KwaZulu-Natal province, South Africa [20]. Although there is evidence that in South Africa XDR TB strains represented at least seven different genotype families [53], all randomly chosen XDR isolates from KwaZulu-Natal which WGS data now is publicly available belong to the same LAM family suggested that one XDR strain had spread throughout the province. The LAM family is known to be rare in European and Asian countries [54]; therefore the attention to MTB strains belonging to other genetic groups, especially drug resistant ones will dramatically extend our knowledge of the TB features.

In the frame of this work, all three MTB strains possessed drug susceptible, MDR and XDR phenotype and picked up for WGS were found to belong to three different lineage. According to the simple classification by *katG463-gyrA95* SNP analysis [42], two of three strains under study (CTRI-2^SENS^ and CTRI-3^MDR^) fall into the principal genetic group 2 (PGG2), while CTRI-4^XDR^ carrying the Arg-Leu polymorphism in the *katG* gene – into PGG 1. SNPs typing based on the combination of 212 nucleotide markers [41] put our CTRI-2^SENS^, CTRI-3^MDR^, and CTRI-4^XDR^ isolates in the SCG-5, SCG-3a, and SCG-2 SNP cluster groups, respectively. Among these, SCG-2 is strongly associated with Beijing family and had the high prevalence of resistance to the most of the anti-tuberculosis drugs [55]. Both of these classifications and phylogenetic analysis based on the comparison of overall SNPs dataset of genomes, 29 housekeeping genes or 75 genes of a mycobacterial 3R system, gives the robust grouping of each of our isolates under study in the particular clusters. We provisionally called this groups “CTRI-2^SENS^-“, “CTRI-3^MDR"^-” and “CTRI-4^XDR^-” clusters. In accordance with the excellent classification system for the human-adopted MTBC given by Gagneux with the co-workers [56]–[59] based on the analysis of genomic deletions, also known as large sequence polymorphisms (LSPs) [58], and, later, on the extended MLST scheme, including 89 genes of mycobacterial genome [57], all members of “CTRI-4^XDR^ cluster” - 02_1987, 94_M4241A, 210, HN878, W-148, X122, T85, CCDC5079 and CCDC5180 strains - belongs to the phylogenetic lineage 2, which is well-known East Asian/Beijing lineage. Both susceptible CTRI-2^SENS^ and multidrug-resistant CTRI-3^MDR^ strains fall into the Euro-American lineage 4. These two lineages belong to the modern MTBC lineages, and they were believed to spread in the world during relatively recent human history. According to these data, the direct comparison of their sequences was hardly to yield the answer on drug resistance mechanisms.

However, we were lucky enough as the WGS data for drug susceptible and drug resistant TB strains, the members of the same genetic group as CTRI-4^XDR^ sequenced under current investigation, have been recently published [60]. Thus, we could analyze the pattern of mutations that distinguishes our sequenced XDR from other genetically related strains with drug susceptible and MDR phenotypes. The implementation of this approach gave us an opportunity to find unique polymorphisms that could explain the nature of XDR phenotype formation.

It should be noted that the simple analysis of genomic data obtained for CTRI-3^MDR^, and CTRI-4^XDR^ isolates in the light of modern knowledge on the molecular mechanisms of drug resistance formation allowed to elucidate the characteristic phenotypes of these isolates at the genetic basis. Whereas we were looking for additional XDR specific mutations, for further investigation, we applied the approach, which removed from the consideration all single-nucleotide substitutions shared by closely related strains of the same genetic family. It allowed to create a list of 76 non-synonymous polymorphisms that were unique to CTRI-4^XDR^. Expectedly all known mutations conferring the resistance to any first- and second-line drugs fall into this list. During the subsequent comparative analysis of the CTRI-4^XDR^ mutations, we focused on potential mechanisms preceding the acquisition of drug resistance in MTB strains which research is of a major interest, especially in the case of XDR.

The data obtained recently showed potential contribution of bacterial cell redox systems in antibacterial action of bactericidal antibiotics [61]. The main damaging agents in this situation are radicals of fatty acids and chemically reactive molecules containing oxygen, so-called reactive oxygen species (ROS) – superoxide (O_2_•^−^), hydrogen peroxide (H_2_O_2_) and hydroxyl radicals (OH•). Molecular oxygen from media diffuses into the cell and interacts with biomolecules. Particularly, ROS formation is those interactions between O_2_ and proteins like respiratory flavoproteins, which have accessible catalytic redox cofactors within their active sites and participate in electron transfer reactions with O_2_. Additionally, the general mechanism of oxidative stress formation involves tricarboxylic acid (TCA) cycle metabolism and a transient depletion of NADH.

So far, in literature available there is no data about influence of amino acid substitutions in redox system enzymes on action of anti-tuberculosis drugs on MTB cells. However, in case of *Pseudomonas aeruginosa,* it is shown that mutations in TCA cycle metabolism and respiratory electron transport chain components decreased killing of this bacterium by aminoglycoside antibiotics [62].

It is worth to note, that genome sequence of CTRI-4^XDR^ isolate contains distinct nonsynonymous substitutions in genes coding enzymes of ferredoxin and flavin containing reductases as well as TCA cycle enzymes. Thus, we described mutations in flavoprotein fumarate reductase (*frdA* gene), isocitrate dehydrogenase (*icd2* gene), NADH dehydrogenase (*nuoN* gene), and enzyme, responsible for reduced/oxidation state of iron (*trxB2* gene). Probably changes in activity of these enzymes influence the metabolism of mycobacteria and support the formation of resistance. Along with specific changes in genes related to SOS response [63] either seems to be characteristic for whole Beijing group (mutations in *polA* and *recD* genes) [33], [64], or unique for CTRI-4^XDR^ (mutation in *dnaE* gene) it also may possibly serve as a condition for appearance of mutable phenotype, characterized by significant resistance to anti-tuberculosis drugs.

It should be noted that particular mutations in enzymes of a redox system are known to provide resistance to isoniazid and ethambutol due to substitutions in *katG* and *embB* genes, respectively, and the mechanism of their influence is well studied. Mutations found in other enzymes of a redox system, although we do not have direct evidence of alterations in their activities, prove that careful study of regularities in formation of resistance to antibacterial agents in mycobacteria is necessary and actual. Close attention should also be paid to a role of SOS response in so-called induced mutagenesis. It is quite clear, that SOS response and ROS, which induce it, influence the mutational activity of the cell. Non-synonymous replacements found in this study in genes of 3R system and a certain amount of replacements in genes of a redox system on the one-hand may determine decrease in individual sensitivity of mycobacterial cells in case of antibacterial agent’s impact, and on the other hand, increased mutability in MTB and further generation of XDR phenotype.

Under this investigation, we have additionally tested two groups of MDR/XDR strains. The pattern of mutations which were analyzed touched on 13 genes of interest especially cording the enzymes of redox system, TCA cycle and SOS response. All of them were picked up in accordance with CTRI-4^XDR^ genome sequence. Unfortunately we failed to find any XDR-specific mutations within this group. Although the additional XDRs belong to the same Beijing family as is CTRI-4^XDR^ they share no other mutations than in genes of 3R system characteristic for whole family [33], [64]. It seems that in case of CTRI-4^XDR^, we deal with the individual features of this strain.

At this stage of scientific knowledge, we cannot draw a conclusion about the existence of some bias in the formation of XDR phenotype. While the genetic mutations associated with first-line and second-line drug resistant TB have been well described, the efforts to outline molecular character specific for MDR and XDR TB are ongoing. Thus WGS of MDR and XDR TB strains from KwaZulu-Natal revealed novel mutations not previously associated with drug resistance, but a further study determined that the majority of them came from a common ancestor, suggesting that XDR strains can evolve independently without fitness changes or XDR-specific mutations [65].

We hope that the further analysis of newly re-sequenced MTB genomes collected worldwide will give us the inestimable benefits allowing to understand an origin and evolution of the bacterial pathogens, including the evolution of drug resistance. The larger number of mutations observed in CTRI-4^XDR^ strain suggests that it can be a potential mechanism for the evolution of drug resistance in mycobacteria. Future work on systems-level analysis of these strains may throw a more definitive light on this important area.

### Data Access

The genomic sequences of CTRI-2^SENS^ and CTRI-4^XDR^ are located at NCBI database under GenBank accession no. CP002992 and AIIE01000000, respectively. Raw sequence data for CTRI-3^MDR^ have been deposited in the NCBI Sequence Read Archive with Accession Number: SRA051492.

## Supporting Information

Table S1 **Individual SNPs identified for CTRI-2(SENS).**
(XLSX)

Table S2 **Individual SNPs identified for CTRI-3(MDR).**
(XLSX)

Table S3 **Individual SNPs identified for CTRI-4(XDR).**
(XLSX)

Table S4 **Significant mutations characteristic for CTRI-4(XDR) isolate.**
(XLSX)

Table S5 **Individual SNPs identified for KZN-R506(XDR) and KZN-605(XDR).**
(XLSX)

Text S1 **Insertion/deletion polymorphism of CTRI-2^SENS^ genome.**
(DOC)
