# Supplementary material for: Comparative Genomic Analysis of Mycobacterium tuberculosis Drug Resistant Strains from Russia
Source: PLoS One. 2013 Feb 20;8(2):e56577. doi: 10.1371/journal.pone.0056577 (PMC3577857; doi:10.1371/journal.pone.0056577)
Supplement: Text S1 — Insertion/deletion polymorphism of CTRI-2SENS genome. (DOC) [file pone.0056577.s006.doc]

**Text S1. Insertion/deletion polymorphism of CTRI-2^SENS^ genome.**

A small amount of indels was found in CTRI-2^SENS^ with respect to H37Rv. In total, CTRI-2^SENS^ contained 100 insertions, 39 of them were fewer than 10 bp. Only 15 insertions appeared to be greater than 1000 bp. Twelve of such “large” insertions are associated with IS6110 insertion sequences, which are an active family of transposons. Five IS6110 insertions occurred in ORF’s: polyketide synthase pks9 (Rv1664), PPE family protein (Rv1800), membrane-associated phospholipase C (Rv2351c), phosphatase Rv3113, and hypothetical protein Rv1550c. Overall, there were 15 copies of IS6110 in CTRI-2^SENS^ as compared to 16 copies in the H37Rv genome, but only three IS6110 sites were positioned in the same places both in H37Rv and CTRI-2^SENS^. One of these three sites is located in the direct repeats (DR) region of H37Rv; surprisingly, our CTRI-2^SENS^ strain contained two IS6110 sequences in this region instead of one – insertion sequence between the spacers 25 and 20 is common for CTRI-2^SENS^ and H37Rv, while IS6110 copy between the spacers 15 and 14 is unique for our strain.

Besides the insertions associated with IS6110, large sequence fragments were found in CTRI-2^SENS^ which are lacking in H37Rv (Table S1). However, all of these sequences have been discovered in F11 or CDC1551 genomes.

104 deletions relative to strain H37Rv were found out in CTRI-2^SENS^. 40 deletions were fewer than 10 bp, 47 deletions were in range of 10-1000 bp and 17 deletions were greater than 1000 bp. Six large deletions are listed in table S1.

**Table S1.** Large-scale polymorphisms (insertion or deletion of length more than 1000 bp) discovered in CTRI-2^SENS^ isolate and no associated with IS6110. Indels identical to both CDC1551 and F11, are selected by grey. Indels identical to CDC1551 are selected by bold. Indels identical to F11 are selected by black.

| Deletions (n = 6) | | | Insertions (n = 4) | | | |
| --- | --- | --- | --- | --- | --- | --- |
| Position in H37Rv | ORF | Product | Position in CTRI-2^SENS^ | Position in F11 | ORF | Product (according to F11 annotation) |
| 453367 - 455972 | Rv0376c | hypothetical protein | 334409 – 337250 | 334971 - 337812 | TBFG_10283 | PE-PGRS family protein |
|  | Rv0377 | LysR family transcriptional regulator |  |  | TBFG_10284 | PE-PGRS family protein |
|  | Rv0378 | glycine rich protein |  |  | TBFG_10285 | PE-PGRS family protein |
| 1779243 - 1788513 | Rv1573-Rv1585 | phiRV1 phage proteins | 1987509 - 1988461 | 2002082 - 2003034* | TBFG_11789 | hypothetical protein |
|  | Rv1586c | phiRv1 integrase |  |  | TBFG_11790 | hypothetical protein |
| 1986626 - 1987702 | Rv1754c | hypothetical protein | 2262290 - 2268647 | 2278338 - 2283337* | TBFG_12059 | hypothetical protein |
|  | Rv1755c | phospholipase C 4 PLCD |  |  | TBFG_12060 | hypothetical protein |
| 2635577 - 2638997 | Rv2353c | PPE family protein |  | Position in CDC1551 |  | Product (according to CDC1551 annotation) |
|  | Rv2354 | transposase | **3704771 - 3710210** | **3705270 - 3709351** | **MT3426** | **pterin-4-alpha-carbinolamine dehydratase** |
|  | Rv2355 | transposase |  |  | **MT3427** | **molybdopterin cofactor biosynthesis protein A** |
|  | Rv2356c | PPE family protein |  |  | **MT3427.1** | **hypothetical protein** |
| **3842767 - 3847692** | **Rv3426** | **PPE family protein** |  |  | **MT3428** | **AfsR/DnrI/RedD family transcriptional regulator** |
|  | **Rv3427c** | **transposase** |  |  | **MT3429** | **hypothetical protein** |
|  | **Rv3428c** | **transposase** |  |  | **MT3427.1** | **hypothetical protein** |
| 3945959 - 3950163 | Rv3514 | PE-PGRS family protein |  | | | |

* - according to F11 annotation
